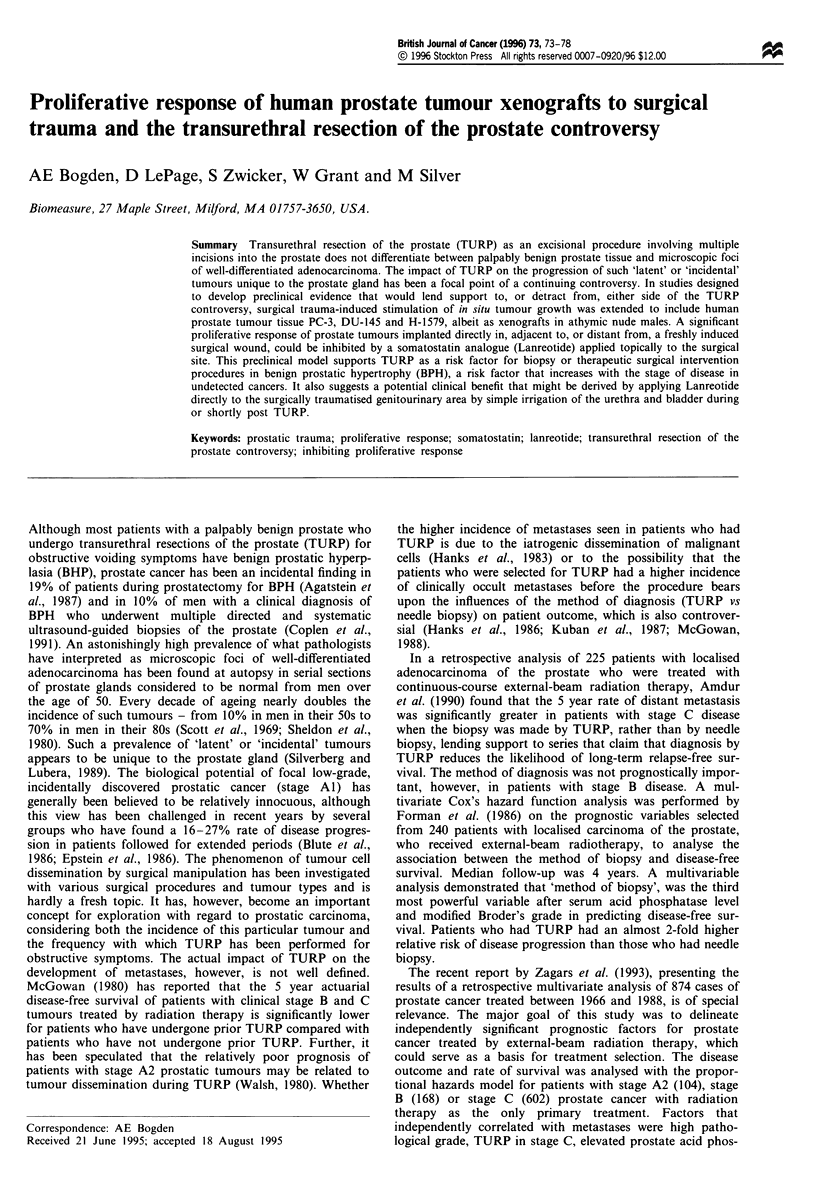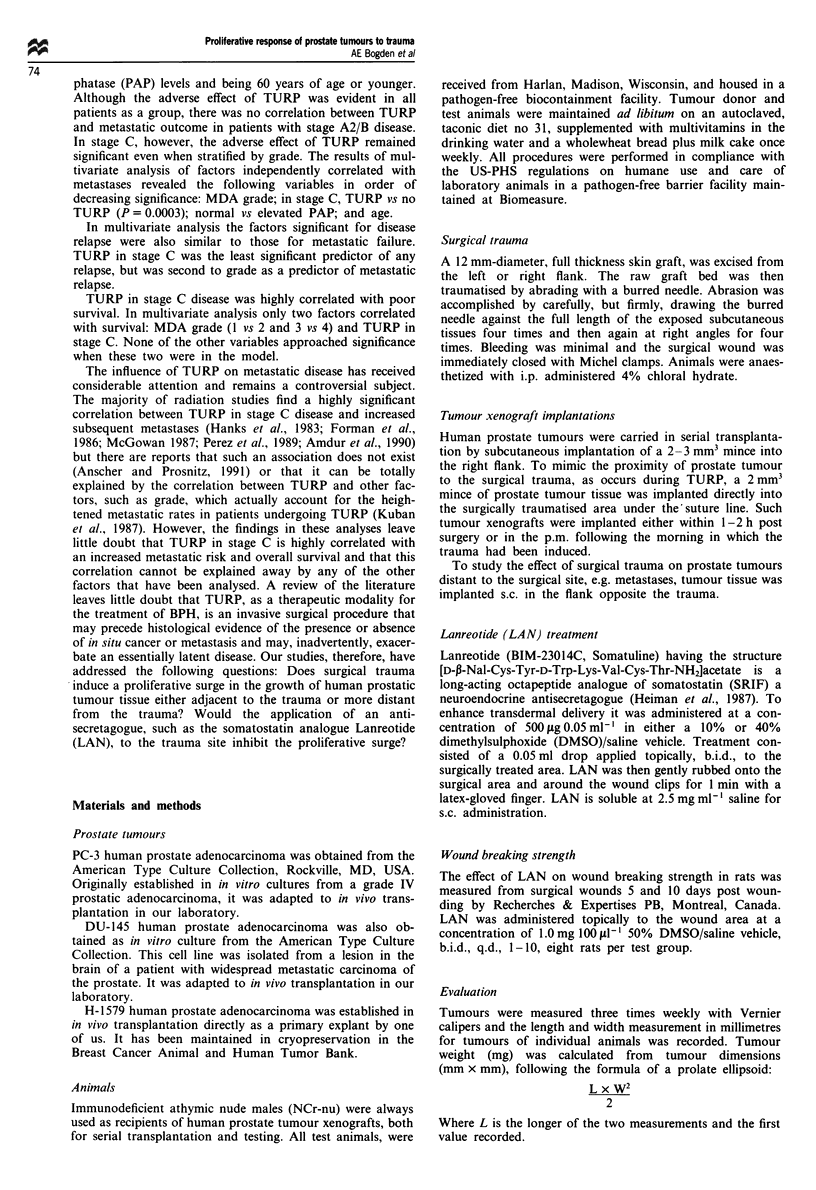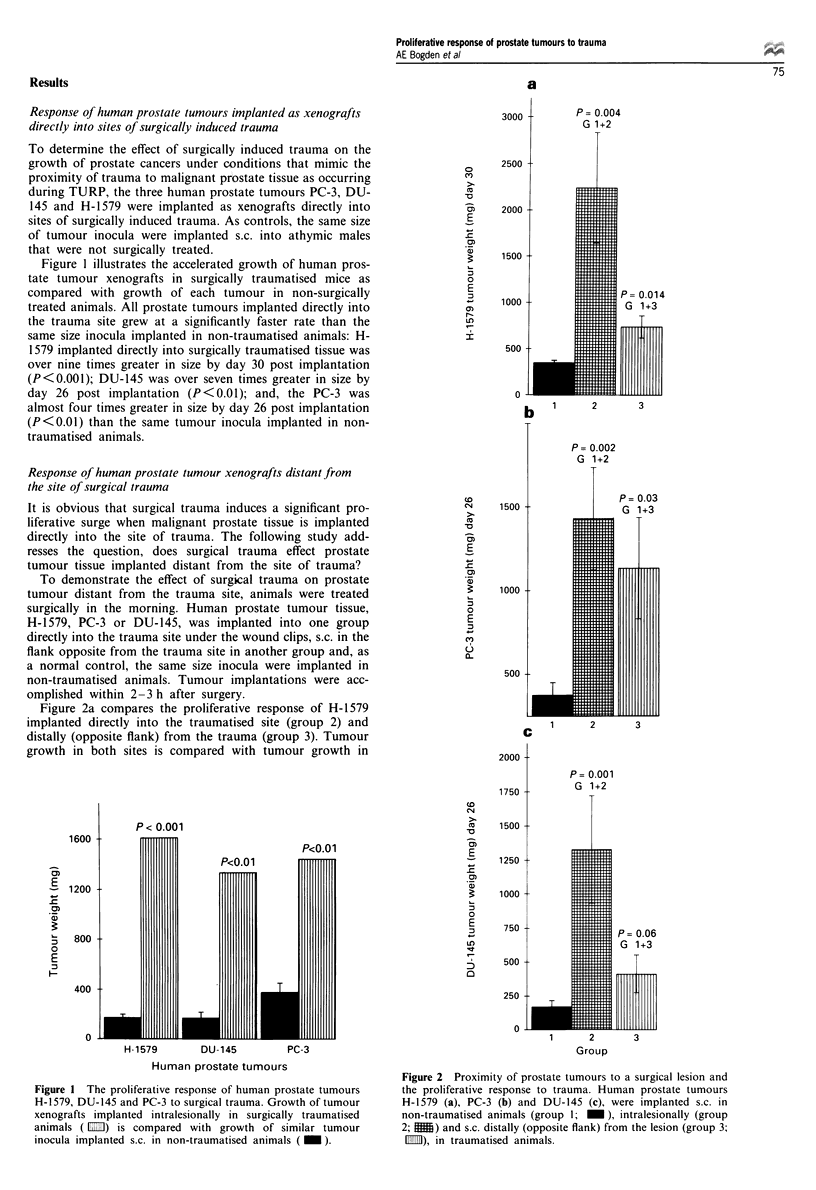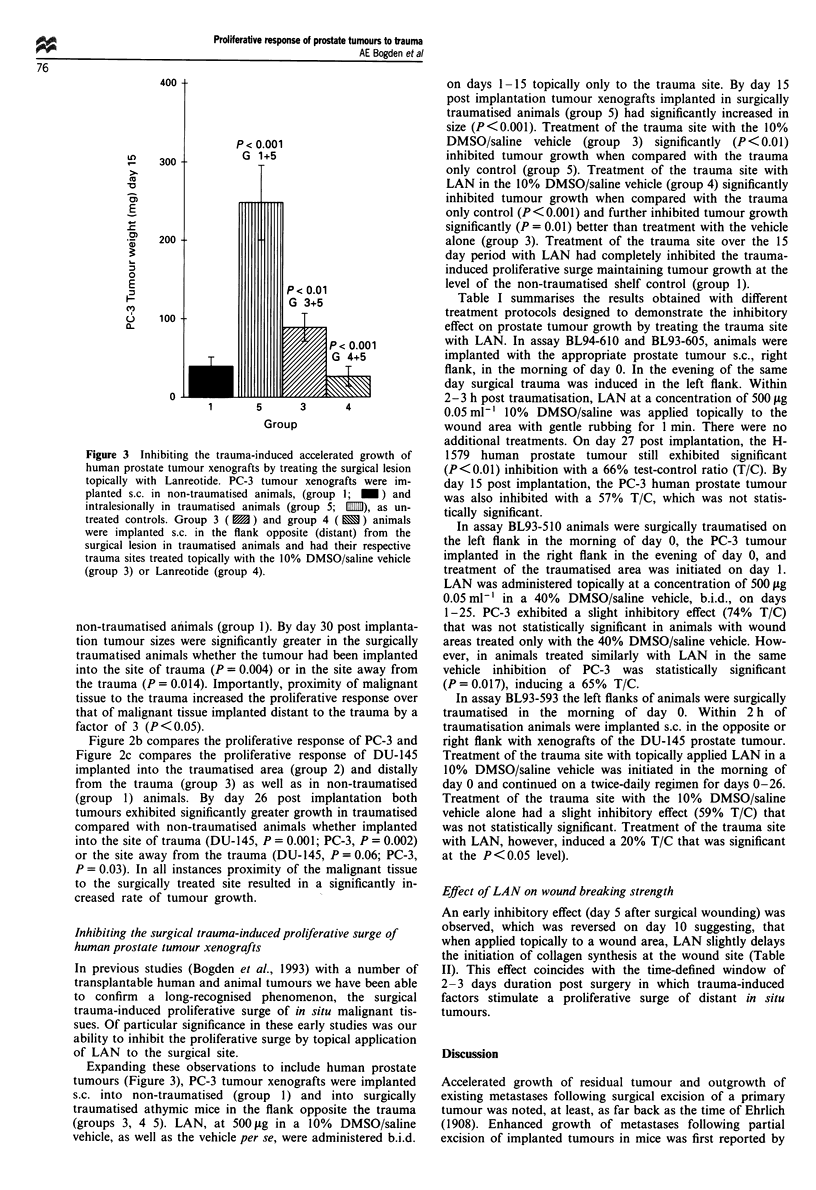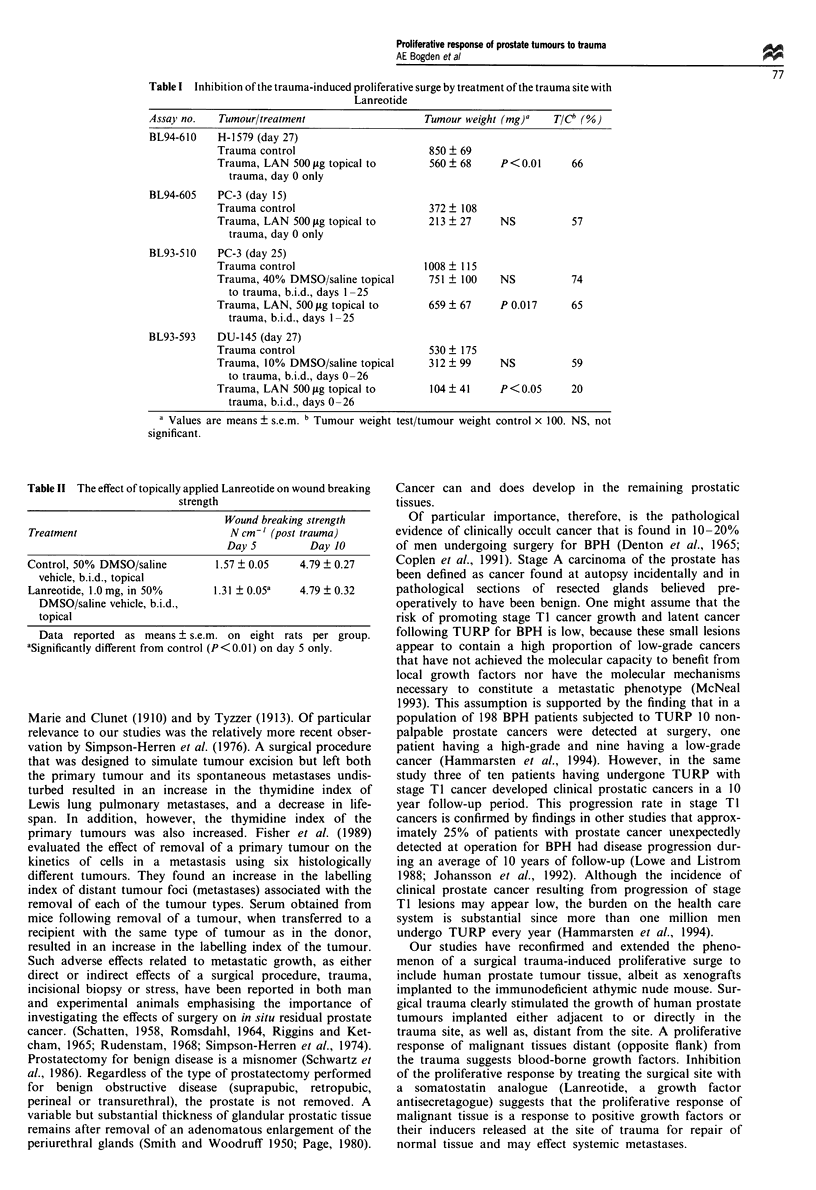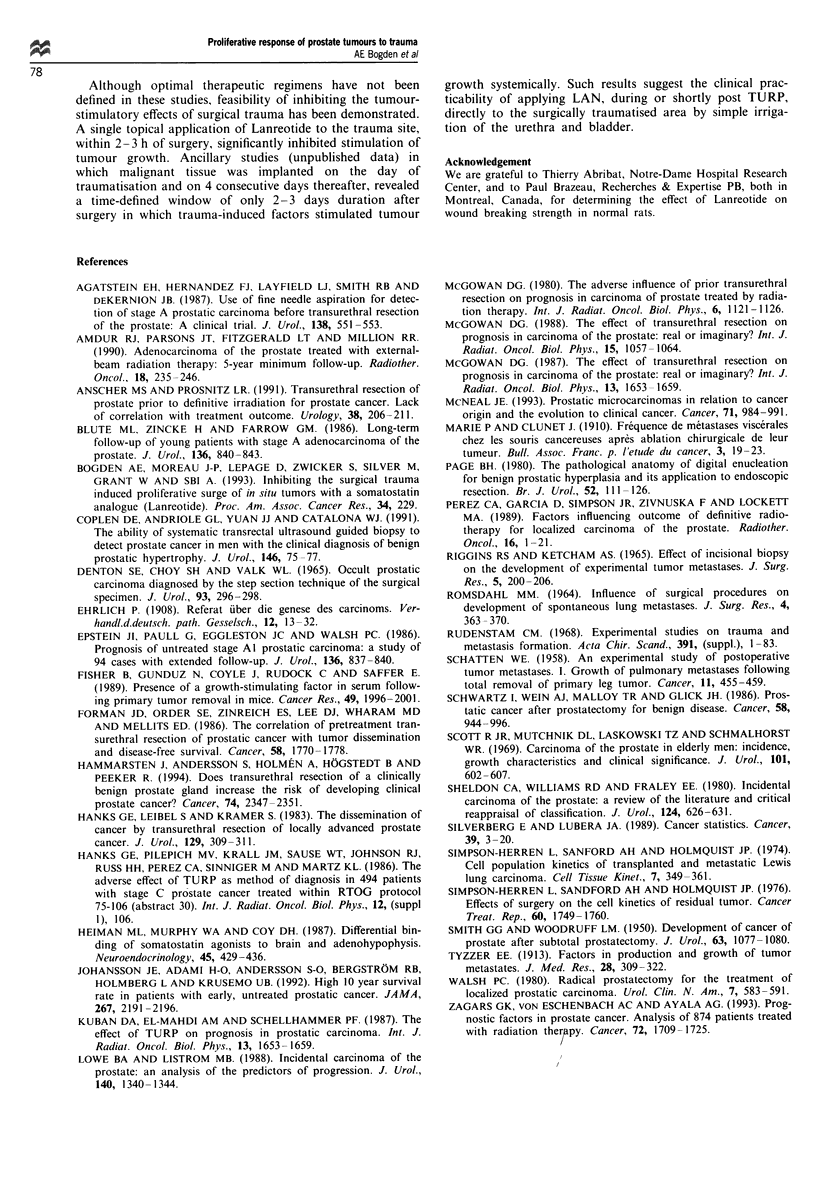# Proliferative response of human prostate tumour xenografts to surgical trauma and the transurethral resection of the prostate controversy.

**DOI:** 10.1038/bjc.1996.13

**Published:** 1996-01

**Authors:** A. E. Bogden, D. LePage, S. Zwicker, W. Grant, M. Silver

**Affiliations:** Biomeasure, Milford, MA 01757-3650, USA.

## Abstract

Transurethral resection of the prostate (TURP) as an excisional procedure involving multiple incisions into the prostate does not differentiate between palpably benign prostate tissue and microscopic foci of well-differentiated adenocarcinoma. The impact of TURP on the progression of such 'latent' or 'incidental' tumours unique to the prostate gland has been a focal point of a continuing controversy. In studies designed to develop preclinical evidence that would lend support to, or detract from, either side of the TURP controversy, surgical trauma-induced stimulation of in situ tumour growth was extended to include human prostate tumour tissue PC-3, DU-145 and H-1579, albeit as xenografts in athymic nude males. A significant proliferative response of prostate tumours implanted directly in, adjacent to, or distant from, a freshly induced surgical wound, could be inhibited by a somatostatin analogue (Lanreotide) applied topically to the surgical site. This preclinical model supports TURP as a risk factor for biopsy or therapeutic surgical intervention procedures in benign prostatic hypertrophy (BPH), a risk factor that increases with the stage of disease in undetected cancers. It also suggests a potential clinical benefit that might be derived by applying Lanreotide directly to the surgically traumatised genitourinary area by simple irrigation of the urethra and bladder during or shortly post TURP.


British Journal of Cancer (1996) 73, 73–78
© 1996 Stockton Press All rights reserved 0007–0920/96 $12.00

### Proliferative response of human prostate tumour xenografts to surgical trauma and the transurethral resection of the prostate controversy

AE Bogden, D LePage, S Zwicker, W Grant and M Silver

*Biomeasure, 27 Maple Street, Milford, MA 01757-3650, USA.*

**Summary** Transurethral resection of the prostate (TURP) as an excisional procedure involving multiple incisions into the prostate does not differentiate between palpably benign prostate tissue and microscopic foci of well-differentiated adenocarcinoma. The impact of TURP on the progression of such 'latent' or 'incidental' tumours unique to the prostate gland has been a focal point of a continuing controversy. In studies designed to develop preclinical evidence that would lend support to, or detract from, either side of the TURP controversy, surgical trauma-induced stimulation of *in situ* tumour growth was extended to include human prostate tumour tissue PC-3, DU-145 and H-1579, albeit as xenografts in athymic nude males. A significant proliferative response of prostate tumours implanted directly in, adjacent to, or distant from, a freshly induced surgical wound, could be inhibited by a somatostatin analogue (Lanreotide) applied topically to the surgical site. This preclinical model supports TURP as a risk factor for biopsy or therapeutic surgical intervention procedures in benign prostatic hypertrophy (BPH), a risk factor that increases with the stage of disease in undetected cancers. It also suggests a potential clinical benefit that might be derived by applying Lanreotide directly to the surgically traumatised genitourinary area by simple irrigation of the urethra and bladder during or shortly post TURP.

**Keywords:** prostatic trauma; proliferative response; somatostatin; lanreotide; transurethral resection of the prostate controversy; inhibiting proliferative response

Although most patients with a palpably benign prostate who undergo transurethral resections of the prostate (TURP) for obstructive voiding symptoms have benign prostatic hyperplasia (BHP), prostate cancer has been an incidental finding in 19% of patients during prostatectomy for BPH (Agatstein *et al*., 1987) and in 10% of men with a clinical diagnosis of BPH who underwent multiple directed and systematic ultrasound-guided biopsies of the prostate (Coplen *et al*., 1991). An astonishingly high prevalence of what pathologists have interpreted as microscopic foci of well-differentiated adenocarcinoma has been found at autopsy in serial sections of prostate glands considered to be normal from men over the age of 50. Every decade of ageing nearly doubles the incidence of such tumours – from 10% in men in their 50s to 70% in men in their 80s (Scott *et al*., 1969; Sheldon *et al*., 1980). Such a prevalence of 'latent' or 'incidental' tumours appears to be unique to the prostate gland (Silverberg and Lubera, 1989). The biological potential of focal low-grade, incidentally discovered prostatic cancer (stage A1) has generally been believed to be relatively innocuous, although this view has been challenged in recent years by several groups who have found a 16–27% rate of disease progression in patients followed for extended periods (Blute *et al*., 1986; Epstein *et al*., 1986). The phenomenon of tumour cell dissemination by surgical manipulation has been investigated with various surgical procedures and tumour types and is hardly a fresh topic. It has, however, become an important concept for exploration with regard to prostatic carcinoma, considering both the incidence of this particular tumour and the frequency with which TURP has been performed for obstructive symptoms. The actual impact of TURP on the development of metastases, however, is not well defined. McGowan (1980) has reported that the 5 year actuarial disease-free survival of patients with clinical stage B and C tumours treated by radiation therapy is significantly lower for patients who have undergone prior TURP compared with patients who have not undergone prior TURP. Further, it has been speculated that the relatively poor prognosis of patients with stage A2 prostatic tumours may be related to tumour dissemination during TURP (Walsh, 1980). Whether the higher incidence of metastases seen in patients who had TURP is due to the iatrogenic dissemination of malignant cells (Hanks *et al*., 1983) or to the possibility that the patients who were selected for TURP had a higher incidence of clinically occult metastases before the procedure bears upon the influences of the method of diagnosis (TURP *vs* needle biopsy) on patient outcome, which is also controversial (Hanks *et al*., 1986; Kuban *et al*., 1987; McGowan, 1988).

In a retrospective analysis of 225 patients with localised adenocarcinoma of the prostate who were treated with continuous-course external-beam radiation therapy, Amdur *et al*. (1990) found that the 5 year rate of distant metastasis was significantly greater in patients with stage C disease when the biopsy was made by TURP, rather than by needle biopsy, lending support to series that claim that diagnosis by TURP reduces the likelihood of long-term relapse-free survival. The method of diagnosis was not prognostically important, however, in patients with stage B disease. A multivariate Cox's hazard function analysis was performed by Forman *et al*. (1986) on the prognostic variables selected from 240 patients with localised carcinoma of the prostate, who received external-beam radiotherapy, to analyse the association between the method of biopsy and disease-free survival. Median follow-up was 4 years. A multivariable analysis demonstrated that 'method of biopsy', was the third most powerful variable after serum acid phosphatase level and modified Broder's grade in predicting disease-free survival. Patients who had TURP had an almost 2-fold higher relative risk of disease progression than those who had needle biopsy.

The recent report by Zagars *et al*. (1993), presenting the results of a retrospective multivariate analysis of 874 cases of prostate cancer treated between 1966 and 1988, is of special relevance. The major goal of this study was to delineate independently significant prognostic factors for prostate cancer treated by external-beam radiation therapy, which could serve as a basis for treatment selection. The disease outcome and rate of survival was analysed with the proportional hazards model for patients with stage A2 (104), stage B (168) or stage C (602) prostate cancer with radiation therapy as the only primary treatment. Factors that independently correlated with metastases were high pathological grade, TURP in stage C, elevated prostate acid phos-

Correspondence: AE Bogden
Received 21 June 1995; accepted 18 August 1995

phatase (PAP) levels and being 60 years of age or younger. Although the adverse effect of TURP was evident in all patients as a group, there was no correlation between TURP and metastatic outcome in patients with stage A2/B disease. In stage C, however, the adverse effect of TURP remained significant even when stratified by grade. The results of multivariate analysis of factors independently correlated with metastases revealed the following variables in order of decreasing significance: MDA grade; in stage C, TURP *vs* no TURP ($P = 0.0003$); normal *vs* elevated PAP; and age.

In multivariate analysis the factors significant for disease relapse were also similar to those for metastatic failure. TURP in stage C was the least significant predictor of any relapse, but was second to grade as a predictor of metastatic relapse.

TURP in stage C disease was highly correlated with poor survival. In multivariate analysis only two factors correlated with survival: MDA grade (1 *vs* 2 and 3 *vs* 4) and TURP in stage C. None of the other variables approached significance when these two were in the model.

The influence of TURP on metastatic disease has received considerable attention and remains a controversial subject. The majority of radiation studies find a highly significant correlation between TURP in stage C disease and increased subsequent metastases (Hanks *et al.*, 1983; Forman *et al.*, 1986; McGowan 1987; Perez *et al.*, 1989; Amdur *et al.*, 1990) but there are reports that such an association does not exist (Anscher and Prosnitz, 1991) or that it can be totally explained by the correlation between TURP and other factors, such as grade, which actually account for the heightened metastatic rates in patients undergoing TURP (Kuban *et al.*, 1987). However, the findings in these analyses leave little doubt that TURP in stage C is highly correlated with an increased metastatic risk and overall survival and that this correlation cannot be explained away by any of the other factors that have been analysed. A review of the literature leaves little doubt that TURP, as a therapeutic modality for the treatment of BPH, is an invasive surgical procedure that may precede histological evidence of the presence or absence of *in situ* cancer or metastasis and may, inadvertently, exacerbate an essentially latent disease. Our studies, therefore, have addressed the following questions: Does surgical trauma induce a proliferative surge in the growth of human prostatic tumour tissue either adjacent to the trauma or more distant from the trauma? Would the application of an antisecretagogue, such as the somatostatin analogue Lanreotide (LAN), to the trauma site inhibit the proliferative surge?

### Materials and methods

### *Prostate tumours*

PC-3 human prostate adenocarcinoma was obtained from the American Type Culture Collection, Rockville, MD, USA. Originally established in *in vitro* cultures from a grade IV prostatic adenocarcinoma, it was adapted to *in vivo* transplantation in our laboratory.

DU-145 human prostate adenocarcinoma was also obtained as *in vitro* culture from the American Type Culture Collection. This cell line was isolated from a lesion in the brain of a patient with widespread metastatic carcinoma of the prostate. It was adapted to *in vivo* transplantation in our laboratory.

H-1579 human prostate adenocarcinoma was established in *in vivo* transplantation directly as a primary explant by one of us. It has been maintained in cryopreservation in the Breast Cancer Animal and Human Tumor Bank.

### *Animals*

Immunodeficient athymic nude males (NCr-nu) were always used as recipients of human prostate tumour xenografts, both for serial transplantation and testing. All test animals, were received from Harlan, Madison, Wisconsin, and housed in a pathogen-free biocontainment facility. Tumour donor and test animals were maintained *ad libitum* on an autoclaved, taconic diet no 31, supplemented with multivitamins in the drinking water and a wholewheat bread plus milk cake once weekly. All procedures were performed in compliance with the US-PHS regulations on humane use and care of laboratory animals in a pathogen-free barrier facility maintained at Biomeasure.

### *Surgical trauma*

A 12 mm-diameter, full thickness skin graft, was excised from the left or right flank. The raw graft bed was then traumatised by abrading with a burred needle. Abrasion was accomplished by carefully, but firmly, drawing the burred needle against the full length of the exposed subcutaneous tissues four times and then again at right angles for four times. Bleeding was minimal and the surgical wound was immediately closed with Michel clamps. Animals were anaesthetized with i.p. administered 4% chloral hydrate.

### *Tumour xenograft implantations*

Human prostate tumours were carried in serial transplantation by subcutaneous implantation of a 2–3 $mm^3$ mince into the right flank. To mimic the proximity of prostate tumour to the surgical trauma, as occurs during TURP, a 2 $mm^3$ mince of prostate tumour tissue was implanted directly into the surgically traumatised area under the suture line. Such tumour xenografts were implanted either within 1–2 h post surgery or in the p.m. following the morning in which the trauma had been induced.

To study the effect of surgical trauma on prostate tumours distant to the surgical site, e.g. metastases, tumour tissue was implanted s.c. in the flank opposite the trauma.

### *Lanreotide (LAN) treatment*

Lanreotide (BIM-23014C, Somatuline) having the structure [D-β-Nal-Cys-Tyr-D-Trp-Lys-Val-Cys-Thr-$NH_2$]acetate is a long-acting octapeptide analogue of somatostatin (SRIF) a neuroendocrine antisecretagogue (Heiman *et al.*, 1987). To enhance transdermal delivery it was administered at a concentration of 500 μg 0.05 $ml^{-1}$ in either a 10% or 40% dimethylsulphoxide (DMSO)/saline vehicle. Treatment consisted of a 0.05 ml drop applied topically, b.i.d., to the surgically treated area. LAN was then gently rubbed onto the surgical area and around the wound clips for 1 min with a latex-gloved finger. LAN is soluble at 2.5 mg $ml^{-1}$ saline for s.c. administration.

### *Wound breaking strength*

The effect of LAN on wound breaking strength in rats was measured from surgical wounds 5 and 10 days post wounding by Recherches & Expertises PB, Montreal, Canada. LAN was administered topically to the wound area at a concentration of 1.0 mg 100 $\mu l^{-1}$ 50% DMSO/saline vehicle, b.i.d., q.d., 1–10, eight rats per test group.

### *Evaluation*

Tumours were measured three times weekly with Vernier calipers and the length and width measurement in millimetres for tumours of individual animals was recorded. Tumour weight (mg) was calculated from tumour dimensions (mm × mm), following the formula of a prolate ellipsoid:

$$\frac{L \times W^2}{2}$$

Where $L$ is the longer of the two measurements and the first value recorded.

### Results

*Response of human prostate tumours implanted as xenografts directly into sites of surgically induced trauma*

To determine the effect of surgically induced trauma on the growth of prostate cancers under conditions that mimic the proximity of trauma to malignant prostate tissue as occurring during TURP, the three human prostate tumours PC-3, DU-145 and H-1579 were implanted as xenografts directly into sites of surgically induced trauma. As controls, the same size of tumour inocula were implanted s.c. into athymic males that were not surgically treated.

Figure 1 illustrates the accelerated growth of human prostate tumour xenografts in surgically traumatised mice as compared with growth of each tumour in non-surgically treated animals. All prostate tumours implanted directly into the trauma site grew at a significantly faster rate than the same size inocula implanted in non-traumatised animals: H-1579 implanted directly into surgically traumatised tissue was over nine times greater in size by day 30 post implantation ($P<0.001$); DU-145 was over seven times greater in size by day 26 post implantation ($P<0.01$); and, the PC-3 was almost four times greater in size by day 26 post implantation ($P<0.01$) than the same tumour inocula implanted in non-traumatised animals.

*Response of human prostate tumour xenografts distant from the site of surgical trauma*

It is obvious that surgical trauma induces a significant proliferative surge when malignant prostate tissue is implanted directly into the site of trauma. The following study addresses the question, does surgical trauma effect prostate tumour tissue implanted distant from the site of trauma?

To demonstrate the effect of surgical trauma on prostate tumour distant from the trauma site, animals were treated surgically in the morning. Human prostate tumour tissue, H-1579, PC-3 or DU-145, was implanted into one group directly into the trauma site under the wound clips, s.c. in the flank opposite from the trauma site in another group and, as a normal control, the same size inocula were implanted in non-traumatised animals. Tumour implantations were accomplished within 2–3 h after surgery.

Figure 2a compares the proliferative response of H-1579 implanted directly into the traumatised site (group 2) and distally (opposite flank) from the trauma (group 3). Tumour growth in both sites is compared with tumour growth in

**Figure 1** The proliferative response of human prostate tumours H-1579, DU-145 and PC-3 to surgical trauma. Growth of tumour xenografts implanted intralesionally in surgically traumatised animals (▥) is compared with growth of similar tumour inocula implanted s.c. in non-traumatised animals (■).

**Figure 2** Proximity of prostate tumours to a surgical lesion and the proliferative response to trauma. Human prostate tumours H-1579 (**a**), PC-3 (**b**) and DU-145 (**c**), were implanted s.c. in non-traumatised animals (group 1; ■), intralesionally (group 2; ▦) and s.c. distally (opposite flank) from the lesion (group 3; ▥), in traumatised animals.

**Figure 3** Inhibiting the trauma-induced accelerated growth of human prostate tumour xenografts by treating the surgical lesion topically with Lanreotide. PC-3 tumour xenografts were implanted s.c. in non-traumatised animals, (group 1; ■) and intralesionally in traumatised animals (group 5; ▥), as untreated controls. Group 3 ( ▨ ) and group 4 ( ▧ ) animals were implanted s.c. in the flank opposite (distant) from the surgical lesion in traumatised animals and had their respective trauma sites treated topically with the 10% DMSO/saline vehicle (group 3) or Lanreotide (group 4).

non-traumatised animals (group 1). By day 30 post implantation tumour sizes were significantly greater in the surgically traumatised animals whether the tumour had been implanted into the site of trauma ($P = 0.004$) or in the site away from the trauma ($P = 0.014$). Importantly, proximity of malignant tissue to the trauma increased the proliferative response over that of malignant tissue implanted distant to the trauma by a factor of 3 ($P < 0.05$).

Figure 2b compares the proliferative response of PC-3 and Figure 2c compares the proliferative response of DU-145 implanted into the traumatised area (group 2) and distally from the trauma (group 3) as well as in non-traumatised (group 1) animals. By day 26 post implantation both tumours exhibited significantly greater growth in traumatised compared with non-traumatised animals whether implanted into the site of trauma (DU-145, $P = 0.001$; PC-3, $P = 0.002$) or the site away from the trauma (DU-145, $P = 0.06$; PC-3, $P = 0.03$). In all instances proximity of the malignant tissue to the surgically treated site resulted in a significantly increased rate of tumour growth.

*Inhibiting the surgical trauma-induced proliferative surge of human prostate tumour xenografts*

In previous studies (Bogden *et al.*, 1993) with a number of transplantable human and animal tumours we have been able to confirm a long-recognised phenomenon, the surgical trauma-induced proliferative surge of *in situ* malignant tissues. Of particular significance in these early studies was our ability to inhibit the proliferative surge by topical application of LAN to the surgical site.

Expanding these observations to include human prostate tumours (Figure 3), PC-3 tumour xenografts were implanted s.c. into non-traumatised (group 1) and into surgically traumatised athymic mice in the flank opposite the trauma (groups 3, 4 5). LAN, at 500 μg in a 10% DMSO/saline vehicle, as well as the vehicle *per se*, were administered b.i.d. on days 1–15 topically only to the trauma site. By day 15 post implantation tumour xenografts implanted in surgically traumatised animals (group 5) had significantly increased in size ($P < 0.001$). Treatment of the trauma site with the 10% DMSO/saline vehicle (group 3) significantly ($P < 0.01$) inhibited tumour growth when compared with the trauma only control (group 5). Treatment of the trauma site with LAN in the 10% DMSO/saline vehicle (group 4) significantly inhibited tumour growth when compared with the trauma only control ($P < 0.001$) and further inhibited tumour growth significantly ($P = 0.01$) better than treatment with the vehicle alone (group 3). Treatment of the trauma site over the 15 day period with LAN had completely inhibited the trauma-induced proliferative surge maintaining tumour growth at the level of the non-traumatised shelf control (group 1).

Table I summarises the results obtained with different treatment protocols designed to demonstrate the inhibitory effect on prostate tumour growth by treating the trauma site with LAN. In assay BL94-610 and BL93-605, animals were implanted with the appropriate prostate tumour s.c., right flank, in the morning of day 0. In the evening of the same day surgical trauma was induced in the left flank. Within 2–3 h post traumatisation, LAN at a concentration of 500 μg 0.05 $ml^{-1}$ 10% DMSO/saline was applied topically to the wound area with gentle rubbing for 1 min. There were no additional treatments. On day 27 post implantation, the H-1579 human prostate tumour still exhibited significant ($P < 0.01$) inhibition with a 66% test-control ratio (T/C). By day 15 post implantation, the PC-3 human prostate tumour was also inhibited with a 57% T/C, which was not statistically significant.

In assay BL93-510 animals were surgically traumatised on the left flank in the morning of day 0, the PC-3 tumour implanted in the right flank in the evening of day 0, and treatment of the traumatised area was initiated on day 1. LAN was administered topically at a concentration of 500 μg 0.05 $ml^{-1}$ in a 40% DMSO/saline vehicle, b.i.d., on days 1–25. PC-3 exhibited a slight inhibitory effect (74% T/C) that was not statistically significant in animals with wound areas treated only with the 40% DMSO/saline vehicle. However, in animals treated similarly with LAN in the same vehicle inhibition of PC-3 was statistically significant ($P = 0.017$), inducing a 65% T/C.

In assay BL93-593 the left flanks of animals were surgically traumatised in the morning of day 0. Within 2 h of traumatisation animals were implanted s.c. in the opposite or right flank with xenografts of the DU-145 prostate tumour. Treatment of the trauma site with topically applied LAN in a 10% DMSO/saline vehicle was initiated in the morning of day 0 and continued on a twice-daily regimen for days 0–26. Treatment of the trauma site with the 10% DMSO/saline vehicle alone had a slight inhibitory effect (59% T/C) that was not statistically significant. Treatment of the trauma site with LAN, however, induced a 20% T/C that was significant at the $P < 0.05$ level).

*Effect of LAN on wound breaking strength*

An early inhibitory effect (day 5 after surgical wounding) was observed, which was reversed on day 10 suggesting, that when applied topically to a wound area, LAN slightly delays the initiation of collagen synthesis at the wound site (Table II). This effect coincides with the time-defined window of 2–3 days duration post surgery in which trauma-induced factors stimulate a proliferative surge of distant *in situ* tumours.

### Discussion

Accelerated growth of residual tumour and outgrowth of existing metastases following surgical excision of a primary tumour was noted, at least, as far back as the time of Ehrlich (1908). Enhanced growth of metastases following partial excision of implanted tumours in mice was first reported by

**Table I** Inhibition of the trauma-induced proliferative surge by treatment of the trauma site with Lanreotide

| Assay no. | Tumour/treatment | Tumour weight (mg)[a] | | T/C[b] (%) |
|---|---|---|---|---|
| BL94-610 | H-1579 (day 27) | | | |
| | Trauma control | 850 ± 69 | | |
| | Trauma, LAN 500 μg topical to trauma, day 0 only | 560 ± 68 | $P<0.01$ | 66 |
| BL94-605 | PC-3 (day 15) | | | |
| | Trauma control | 372 ± 108 | | |
| | Trauma, LAN 500 μg topical to trauma, day 0 only | 213 ± 27 | NS | 57 |
| BL93-510 | PC-3 (day 25) | | | |
| | Trauma control | 1008 ± 115 | | |
| | Trauma, 40% DMSO/saline topical to trauma, b.i.d., days 1–25 | 751 ± 100 | NS | 74 |
| | Trauma, LAN, 500 μg topical to trauma, b.i.d., days 1–25 | 659 ± 67 | $P$ 0.017 | 65 |
| BL93-593 | DU-145 (day 27) | | | |
| | Trauma control | 530 ± 175 | | |
| | Trauma, 10% DMSO/saline topical to trauma, b.i.d., days 0–26 | 312 ± 99 | NS | 59 |
| | Trauma, LAN 500 μg topical to trauma, b.i.d., days 0–26 | 104 ± 41 | $P<0.05$ | 20 |

[a] Values are means ± s.e.m. [b] Tumour weight test/tumour weight control × 100. NS, not significant.

**Table II** The effect of topically applied Lanreotide on wound breaking strength

| Treatment | Wound breaking strength N cm$^{-1}$ (post trauma) Day 5 | Day 10 |
|---|---|---|
| Control, 50% DMSO/saline vehicle, b.i.d., topical | 1.57 ± 0.05 | 4.79 ± 0.27 |
| Lanreotide, 1.0 mg, in 50% DMSO/saline vehicle, b.i.d., topical | 1.31 ± 0.05[a] | 4.79 ± 0.32 |

Data reported as means ± s.e.m. on eight rats per group. [a]Significantly different from control ($P<0.01$) on day 5 only.

Marie and Clunet (1910) and by Tyzzer (1913). Of particular relevance to our studies was the relatively more recent observation by Simpson-Herren *et al.* (1976). A surgical procedure that was designed to simulate tumour excision but left both the primary tumour and its spontaneous metastases undisturbed resulted in an increase in the thymidine index of Lewis lung pulmonary metastases, and a decrease in lifespan. In addition, however, the thymidine index of the primary tumours was also increased. Fisher *et al.* (1989) evaluated the effect of removal of a primary tumour on the kinetics of cells in a metastasis using six histologically different tumours. They found an increase in the labelling index of distant tumour foci (metastases) associated with the removal of each of the tumour types. Serum obtained from mice following removal of a tumour, when transferred to a recipient with the same type of tumour as in the donor, resulted in an increase in the labelling index of the tumour. Such adverse effects related to metastatic growth, as either direct or indirect effects of a surgical procedure, trauma, incisional biopsy or stress, have been reported in both man and experimental animals emphasising the importance of investigating the effects of surgery on *in situ* residual prostate cancer. (Schatten, 1958, Romsdahl, 1964, Riggins and Ketcham, 1965; Rudenstam, 1968; Simpson-Herren *et al.*, 1974). Prostatectomy for benign disease is a misnomer (Schwartz *et al.*, 1986). Regardless of the type of prostatectomy performed for benign obstructive disease (suprapubic, retropubic, perineal or transurethral), the prostate is not removed. A variable but substantial thickness of glandular prostatic tissue remains after removal of an adenomatous enlargement of the periurethral glands (Smith and Woodruff 1950; Page, 1980). Cancer can and does develop in the remaining prostatic tissues.

Of particular importance, therefore, is the pathological evidence of clinically occult cancer that is found in 10–20% of men undergoing surgery for BPH (Denton *et al.*, 1965; Coplen *et al.*, 1991). Stage A carcinoma of the prostate has been defined as cancer found at autopsy incidentally and in pathological sections of resected glands believed preoperatively to have been benign. One might assume that the risk of promoting stage T1 cancer growth and latent cancer following TURP for BPH is low, because these small lesions appear to contain a high proportion of low-grade cancers that have not achieved the molecular capacity to benefit from local growth factors nor have the molecular mechanisms necessary to constitute a metastatic phenotype (McNeal 1993). This assumption is supported by the finding that in a population of 198 BPH patients subjected to TURP 10 nonpalpable prostate cancers were detected at surgery, one patient having a high-grade and nine having a low-grade cancer (Hammarsten *et al.*, 1994). However, in the same study three of ten patients having undergone TURP with stage T1 cancer developed clinical prostatic cancers in a 10 year follow-up period. This progression rate in stage T1 cancers is confirmed by findings in other studies that approximately 25% of patients with prostate cancer unexpectedly detected at operation for BPH had disease progression during an average of 10 years of follow-up (Lowe and Listrom 1988; Johansson *et al.*, 1992). Although the incidence of clinical prostate cancer resulting from progression of stage T1 lesions may appear low, the burden on the health care system is substantial since more than one million men undergo TURP every year (Hammarsten *et al.*, 1994).

Our studies have reconfirmed and extended the phenomenon of a surgical trauma-induced proliferative surge to include human prostate tumour tissue, albeit as xenografts implanted to the immunodeficient athymic nude mouse. Surgical trauma clearly stimulated the growth of human prostate tumours implanted either adjacent to or directly in the trauma site, as well as, distant from the site. A proliferative response of malignant tissues distant (opposite flank) from the trauma suggests blood-borne growth factors. Inhibition of the proliferative response by treating the surgical site with a somatostatin analogue (Lanreotide, a growth factor antisecretagogue) suggests that the proliferative response of malignant tissue is a response to positive growth factors or their inducers released at the site of trauma for repair of normal tissue and may effect systemic metastases.

Although optimal therapeutic regimens have not been defined in these studies, feasibility of inhibiting the tumour-stimulatory effects of surgical trauma has been demonstrated. A single topical application of Lanreotide to the trauma site, within 2–3 h of surgery, significantly inhibited stimulation of tumour growth. Ancillary studies (unpublished data) in which malignant tissue was implanted on the day of traumatisation and on 4 consecutive days thereafter, revealed a time-defined window of only 2–3 days duration after surgery in which trauma-induced factors stimulated tumour growth systemically. Such results suggest the clinical practicability of applying LAN, during or shortly post TURP, directly to the surgically traumatised area by simple irrigation of the urethra and bladder.

**Acknowledgement**

We are grateful to Thierry Abribat, Notre-Dame Hospital Research Center, and to Paul Brazeau, Recherches & Expertise PB, both in Montreal, Canada, for determining the effect of Lanreotide on wound breaking strength in normal rats.

### References

AGATSTEIN EH, HERNANDEZ FJ, LAYFIELD LJ, SMITH RB AND DEKERNION JB. (1987). Use of fine needle aspiration for detection of stage A prostatic carcinoma before transurethral resection of the prostate: A clinical trial. *J. Urol.*, **138**, 551–553.

AMDUR RJ, PARSONS JT, FITZGERALD LT AND MILLION RR. (1990). Adenocarcinoma of the prostate treated with external-beam radiation therapy: 5-year minimum follow-up. *Radiother. Oncol.*, **18**, 235–246.

ANSCHER MS AND PROSNITZ LR. (1991). Transurethral resection of prostate prior to definitive irradiation for prostate cancer. Lack of correlation with treatment outcome. *Urology*, **38**, 206–211.

BLUTE ML, ZINCKE H AND FARROW GM. (1986). Long-term follow-up of young patients with stage A adenocarcinoma of the prostate. *J. Urol.*, **136**, 840–843.

BOGDEN AE, MOREAU J-P, LEPAGE D, ZWICKER S, SILVER M, GRANT W AND SBI A. (1993). Inhibiting the surgical trauma induced proliferative surge of *in situ* tumors with a somatostatin analogue (Lanreotide). *Proc. Am. Assoc. Cancer Res.*, **34**, 229.

COPLEN DE, ANDRIOLE GL, YUAN JJ AND CATALONA WJ. (1991). The ability of systematic transrectal ultrasound guided biopsy to detect prostate cancer in men with the clinical diagnosis of benign prostatic hypertrophy. *J. Urol.*, **146**, 75–77.

DENTON SE, CHOY SH AND VALK WL. (1965). Occult prostatic carcinoma diagnosed by the step section technique of the surgical specimen. *J. Urol.*, **93**, 296–298.

EHRLICH P. (1908). Referat über die genese des carcinoms. *Verhandl.d.deutsch. path. Gesselsch.*, **12**, 13–32.

EPSTEIN JI, PAULL G, EGGLESTON JC AND WALSH PC. (1986). Prognosis of untreated stage A1 prostatic carcinoma: a study of 94 cases with extended follow-up. *J. Urol.*, **136**, 837–840.

FISHER B, GUNDUZ N, COYLE J, RUDOCK C AND SAFFER E. (1989). Presence of a growth-stimulating factor in serum following primary tumor removal in mice. *Cancer Res.*, **49**, 1996–2001.

FORMAN JD, ORDER SE, ZINREICH ES, LEE DJ, WHARAM MD AND MELLITS ED. (1986). The correlation of pretreatment transurethral resection of prostatic cancer with tumor dissemination and disease-free survival. *Cancer*, **58**, 1770–1778.

HAMMARSTEN J, ANDERSSON S, HOLMÉN A, HÖGSTEDT B AND PEEKER R. (1994). Does transurethral resection of a clinically benign prostate gland increase the risk of developing clinical prostate cancer? *Cancer*, **74**, 2347–2351.

HANKS GE, LEIBEL S AND KRAMER S. (1983). The dissemination of cancer by transurethral resection of locally advanced prostate cancer. *J. Urol.*, **129**, 309–311.

HANKS GE, PILEPICH MV, KRALL JM, SAUSE WT, JOHNSON RJ, RUSS HH, PEREZ CA, SINNIGER M AND MARTZ KL. (1986). The adverse effect of TURP as method of diagnosis in 494 patients with stage C prostate cancer treated within RTOG protocol 75-106 (abstract 30). *Int. J. Radiat. Oncol. Biol. Phys.*, **12**, (suppl 1), 106.

HEIMAN ML, MURPHY WA AND COY DH. (1987). Differential binding of somatostatin agonists to brain and adenohypophysis. *Neuroendocrinology*, **45**, 429–436.

JOHANSSON JE, ADAMI H-O, ANDERSSON S-O, BERGSTRÖM RB, HOLMBERG L AND KRUSEMO UB. (1992). High 10 year survival rate in patients with early, untreated prostatic cancer. *JAMA*, **267**, 2191–2196.

KUBAN DA, EL-MAHDI AM AND SCHELLHAMMER PF. (1987). The effect of TURP on prognosis in prostatic carcinoma. *Int. J. Radiat. Oncol. Biol. Phys.*, **13**, 1653–1659.

LOWE BA AND LISTROM MB. (1988). Incidental carcinoma of the prostate: an analysis of the predictors of progression. *J. Urol.*, **140**, 1340–1344.

MCGOWAN DG. (1980). The adverse influence of prior transurethral resection on prognosis in carcinoma of prostate treated by radiation therapy. *Int. J. Radiat. Oncol. Biol. Phys.*, **6**, 1121–1126.

MCGOWAN DG. (1988). The effect of transurethral resection on prognosis in carcinoma of the prostate: real or imaginary? *Int. J. Radiat. Oncol. Biol. Phys.*, **15**, 1057–1064.

MCGOWAN DG. (1987). The effect of transurethral resection on prognosis in carcinoma of the prostate: real or imaginary? *Int. J. Radiat. Oncol. Biol. Phys.*, **13**, 1653–1659.

MCNEAL JE. (1993). Prostatic microcarcinomas in relation to cancer origin and the evolution to clinical cancer. *Cancer*, **71**, 984–991.

MARIE P AND CLUNET J. (1910). Fréquence de métastases viscérales chez les souris cancereuses après ablation chirurgicale de leur tumeur. *Bull. Assoc. Franc. p. l'etude du cancer*, **3**, 19–23.

PAGE BH. (1980). The pathological anatomy of digital enucleation for benign prostatic hyperplasia and its application to endoscopic resection. *Br. J. Urol.*, **52**, 111–126.

PEREZ CA, GARCIA D, SIMPSON JR, ZIVNUSKA F AND LOCKETT MA. (1989). Factors influencing outcome of definitive radiotherapy for localized carcinoma of the prostate. *Radiother. Oncol.*, **16**, 1–21.

RIGGINS RS AND KETCHAM AS. (1965). Effect of incisional biopsy on the development of experimental tumor metastases. *J. Surg. Res.*, **5**, 200–206.

ROMSDAHL MM. (1964). Influence of surgical procedures on development of spontaneous lung metastases. *J. Surg. Res.*, **4**, 363–370.

RUDENSTAM CM. (1968). Experimental studies on trauma and metastasis formation. *Acta Chir. Scand.*, **391**, (suppl.), 1–83.

SCHATTEN WE. (1958). An experimental study of postoperative tumor metastases. I. Growth of pulmonary metastases following total removal of primary leg tumor. *Cancer*, **11**, 455–459.

SCHWARTZ I, WEIN AJ, MALLOY TR AND GLICK JH. (1986). Prostatic cancer after prostatectomy for benign disease. *Cancer*, **58**, 944–996.

SCOTT R JR, MUTCHNIK DL, LASKOWSKI TZ AND SCHMALHORST WR. (1969). Carcinoma of the prostate in elderly men: incidence, growth characteristics and clinical significance. *J. Urol.*, **101**, 602–607.

SHELDON CA, WILLIAMS RD AND FRALEY EE. (1980). Incidental carcinoma of the prostate: a review of the literature and critical reappraisal of classification. *J. Urol.*, **124**, 626–631.

SILVERBERG E AND LUBERA JA. (1989). Cancer statistics. *Cancer*, **39**, 3–20.

SIMPSON-HERREN L, SANFORD AH AND HOLMQUIST JP. (1974). Cell population kinetics of transplanted and metastatic Lewis lung carcinoma. *Cell Tissue Kinet.*, **7**, 349–361.

SIMPSON-HERREN L, SANDFORD AH AND HOLMQUIST JP. (1976). Effects of surgery on the cell kinetics of residual tumor. *Cancer Treat. Rep.*, **60**, 1749–1760.

SMITH GG AND WOODRUFF LM. (1950). Development of cancer of prostate after subtotal prostatectomy. *J. Urol.*, **63**, 1077–1080.

TYZZER EE. (1913). Factors in production and growth of tumor metastates. *J. Med. Res.*, **28**, 309–322.

WALSH PC. (1980). Radical prostatectomy for the treatment of localized prostatic carcinoma. *Urol. Clin. N. Am.*, **7**, 583–591.

ZAGARS GK, VON ESCHENBACH AC AND AYALA AG. (1993). Prognostic factors in prostate cancer. Analysis of 874 patients treated with radiation therapy. *Cancer*, **72**, 1709–1725.